# Abdominoplasty Through Six Decades: A Bibliometric Analysis

**DOI:** 10.1055/s-0045-1814166

**Published:** 2026-01-05

**Authors:** Nada Mohamed Elnegeri, Shorouk Mohsen, Hisham Mostafa Elgendy, Mohammed Hassan El-Fahar, Ahmed Hassan El-Sabbagh

**Affiliations:** 1 Department of Plastic Surgery, Faculty of Medicine, Mansoura University, Mansoura, Egypt; 2 Department of Public Health and Community Medicine, Faculty of Medicine, Mansoura University, Mansoura, Egypt; 3 Department of General Surgery, Faculty of Medicine, Mansoura University, Mansoura, Egypt

**Keywords:** abdominoplasty, global analysis, Arabic-speaking countries, bibliometric analysis, visualization analysis

## Abstract

Abdominoplasty has become a common practice in plastic surgery globally. With the introduction of bariatric surgery and many adjuvant procedures, the number of patients seeking abdominoplasty has increased. Currently, bibliometric analysis is used to analyze the literature deeply to get a comprehensive study. In this study, a bibliometric analysis of abdominoplasty was performed, investigating research patterns both worldwide and among Arab countries. A study was performed using the Web of Science Core Collection database, selecting paper type to Research article or Review article and language to English. Following a review process involving three researchers, we identified the documents to be included in the bibliometric analysis. Subsequently, we employed the Visualization of Similarities Viewer software (VOSviewer), and CiteSpace software to conduct visualization analysis of basic information and trends regarding publications, countries, institutions, journals, authors, citations, keywords, funding, and subject categories. A total of 1,886 relevant articles were selected from 1960 to January 2024. The United States dominates global abdominoplasty research (43%), while Egypt and Saudi Arabia are the leading contributors in the Arab world. University of Pittsburgh (68 publications) and Federal University of São Paulo (31 publications) emerged as highly productive institutions. Within the Arab region, King Saud Medical City ranks first. Plastic and Reconstructive Surgery Journal stands out as the most prestigious journal in this field; 111 authors were recognized to publish at least five publications. This retrieved data comprised 34,781 citations and 3,845 keywords; most of the incorporated publications had no source of funding and were under the surgery category. This study sheds light on the research landscape in abdominoplasty. The data reflect rapid expansion in this subject, with the United States occupying the leading position worldwide. International collaboration is essential and highly recommended. Forthcoming aspects of research may concentrate on new techniques, outcomes, and assessment of quality of life.

## Introduction

Body contouring surgery is a rapidly evolving sector in the realm of plastic surgery. 1 It has been subcategorized according to anatomical region into breast surgery, upper body lift, lower body lift, and abdominoplasty. 2

Modern abdominoplasty dates back to 1960. It is a bona fide body contouring surgery with panniculectomy (excess abdominal skin removal), plication of the abdominal wall, liposuction of flanks, and resetting of the umbilicus. It can be combined with other aesthetic procedures or intra-abdominal and pelvic operations. 3 Abdominoplasty is reserved for nonobese patients as a cosmetic operation, while panniculectomy is a reconstructive procedure to decrease the infection rate and to increase hygiene. 4

Since the 1980s, liposuction has been one of the cornerstones of body contouring procedures. It can be done solely or with other excisional procedures. Many noninvasive alternative techniques to liposuction are available, such as ultrasound, laser, infrared, radiofrequency, and cryolipolysis. 5

With the advent of the 21st century, bariatric surgery has emerged as a major surgical solution for the growing number of obese patients worldwide seeking weight loss. As a result of the boom of bariatric surgery, there is a tremendous increase in the number of patients referred for body contouring, especially abdominoplasty. 6

Abdominoplasty complication ranges between 32 and 37%. The most common complications are seroma, hematoma, and wound dehiscence. 7

Due to the tremendous increase in the number of published articles, it is difficult to make a manual assessment of the topic. 8 This can be achieved by bibliometric analysis. It is an interdisciplinary method that applies mathematical and statistical methods to analyze publications. 9 Bibliometric analysis was highlighted by Pritchard and has been widely applied in scientific literature, including plastic surgery. 10 11

Meta-analyses and systematic reviews are applied to answer a research question. Meanwhile, bibliometrics statistically analyzes information such as annual publications, geographic regions, institutions, journals, references, authors, citations, keywords, and funding sources. 12

Till now, Arab countries remain underrepresented in the production and collaboration of plastic surgery research. There are 22 Arabic-speaking countries, distributed mainly in North Africa and the Middle East. 13

In the absence of a sufficient number of bibliometric publications on abdominoplasty globally and a lack of articles shedding light on the contribution of the Arabic world, a bibliometric analysis study was conducted to assess academic influence, demonstrate the scientific evolution, and emphasize the key features of publications on abdominoplasty over six decades globally and in Arab countries. This information can help identify abdominoplasty research trends in the region, as well as identify prospective collaborations and cooperative research opportunities.

## Materials and Methods

### Search Strategy

After approval from the ethics committee of our university (IRB code: MS.22.12.2253), this bibliometric analysis was conducted. The Web of Science Core Collection (WoSCC) database was used to generate relevant studies on abdominoplasty. The search terms in the database were designed as follows: ((TS = (abdominoplasty)) OR TS = (panniculectomy)) OR TS = (“tummy tuck*”)) OR TS = (“Body Contour*”). The title, abstract, and author keywords were searched. The current study included only English-language documents; the document type was set as “Research article or Review article”; and the publication time span was set from January 1, 1960 to January 31, 2024.

**Fig. 1:**
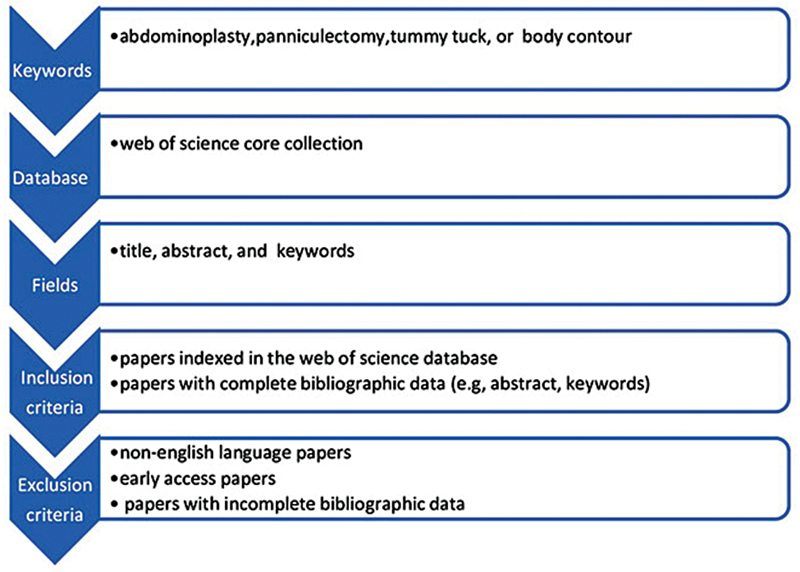
Research flow study diagram.

Two researchers separately reviewed the literature, gathered data, and double-checked the references; a third researcher arbitrated any discrepancies. The search yielded 3,681 articles. These articles were then reviewed to identify if they were associated with abdominoplasty and fulfilled eligibility criteria. Finally, 1,886 articles were identified ( Fig. 1 ). Data on date of publication, countries of focus for these eligible studies, and countries of institutional affiliation of lead and senior authors were extracted and used for the current analysis.

To analyze publication trends in the Arabic-speaking countries, a sub-analysis was conducted that included only articles published from the 22 states of the Arab League: Algeria, Bahrain, Comoros, Djibouti, Egypt, Iraq, Jordan, Kuwait, Lebanon, Libya, Mauritania, Morocco, Oman, Palestinian Authority, Qatar, Saudi Arabia, Somalia, Sudan, Syria, Tunisia, United Arab Emirates, and Yemen. A total of 141 documents were retrieved using this search; some of which were irrelevant to the current topic. As a result, 82 articles were kept for additional analysis.

### Data Analysis and Visualization

VOSviewer (Visualization of Similarities Viewer) is a software tool that creates network-based maps and analyzes bibliometric networks. 14

The VOSviewer software v.1.6.16 (Centre for Science and Technology Studies, Leiden University) was used to analyze and visualize abdominoplasty publications. This included bibliographical information, abstracts, research organizations, countries, keywords, and terms. Co-authorship, keyword co-occurrence analyses, citation, co-cited authors, and journals were used in this study. Co-authorship identifies the most efficient and mutually published documents. A bibliometric network represents the relationships between researchers, research institutions, and countries based on the number of papers they have co-authored together. Co-occurrence of author keywords identifies terms that appear more frequently in publications. CiteSpace is a free Java application for visualizing and analyzing temporal patterns and trends of a scientific field. CiteSpace 5.8.R3 (64-bit) was used to identify top references and keywords with the strongest citation bursts. 15 In addition, an online analytic platform ( http://bibliometric.com/ ) for bibliometric analysis was used.

## Results

### Publications

From 1957 to January 2024, a total of 1,886 publications were identified from the WoSCC. These included original research articles (1,732/1,886, 92%) and review articles (149/1,886, 7.9%). In the Arab region, 82 papers were identified; 69/82 (85.1%) were research articles and 12/82 (14.8%) were review articles.

**Fig. 2:**
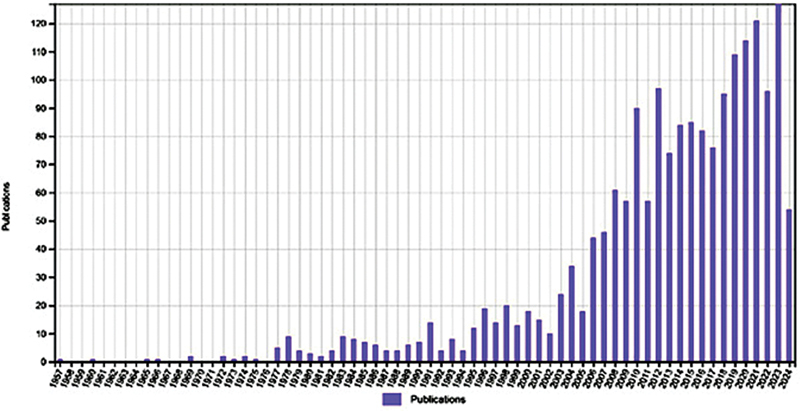
Distribution of published documents of abdominoplasty globally from 1960 to January 2024.

**Fig. 3:**
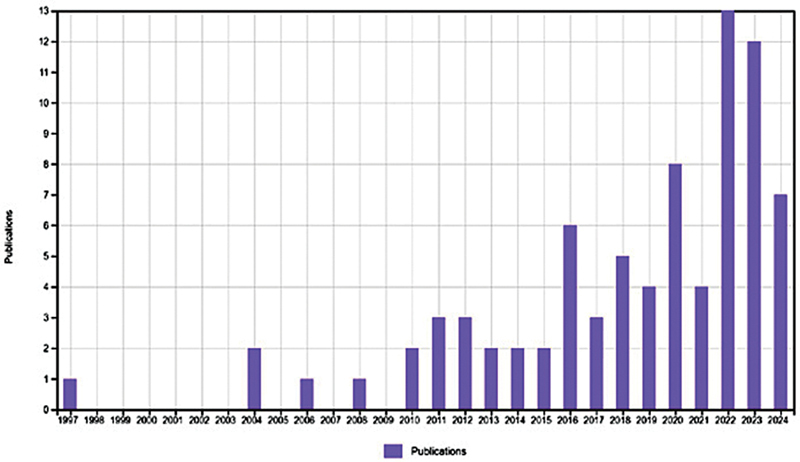
Distribution of published documents of abdominoplasty in Arab countries between 1960 and January 2024.

**Fig. 4:**
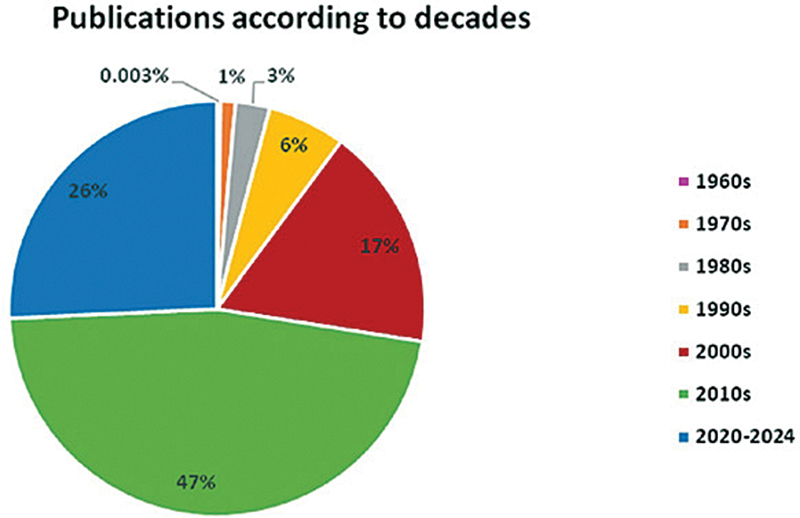
Distribution of abdominoplasty articles according to decades.

The yearly distribution of publications over time is shown in Fig. 2 . Between 1957 and 2007, there was a consistent growth in the number of publications without any distinct research patterns. From 2008 to 2012, there was a significant surge in publications, with minimal fluctuations in the last 2 years. However, there was a drop in the trend of publications between 2013 and 2017. Then a sharp increase was observed from 2018 onwards, and the annual publication output increased significantly in 2023, exceeding more than twice the annual publication output in 2009. In Arab countries, there was a paucity of publications in abdominoplasty till 2010 ( Fig. 3 ). To better understand the growth of this field, we have divided the timeline from 1960 to 2024 into five periods ( Fig. 4 ).

### Countries

Most publications were affiliated mainly with the United States (811/1,886, 43%) and Brazil (137/1,886, 7.2%), followed by England (109/1,886, 5.7%), Italy (105/1,886, 5.5%), and Canada (73/1,886, 4%). In terms of citations, the United States ( *N* = 15,823) was significantly ahead of Brazil ( *N* = 2,217) and Canada ( *N* = 1,617; Table 1 ).

**Table 1:** Top 10 countries publishing abdominoplasty research

Country	Number of publications	Number of citations
**United States**	811	18,078
**Brazil**	137	2,282
**England**	109	1,920
**Italy**	105	1,263
**Canada**	73	1,736
**Germany**	69	1,396
**France**	45	664
**Austria**	35	682
**Netherlands**	33	670
**Egypt**	30	124

**Fig. 5:**
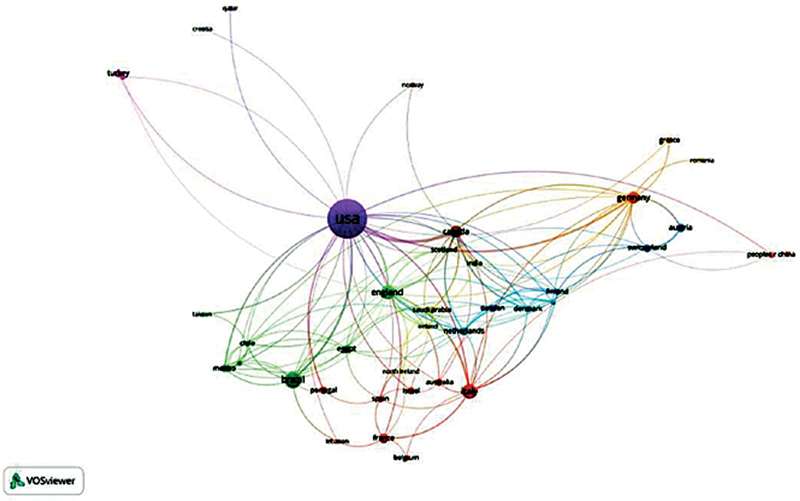
Network visualization map of country co-authorship worldwide.

**Fig. 6:**
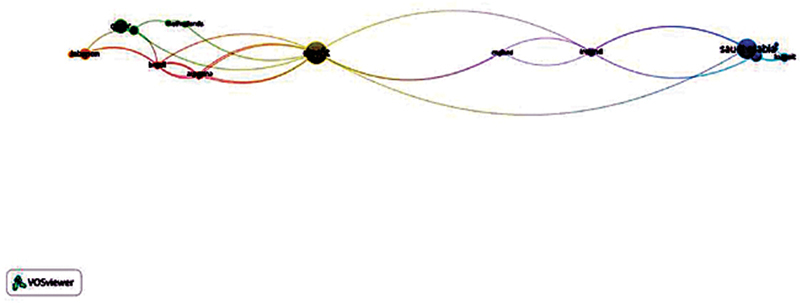
Network visualization map of country co-authorship in Arab countries.

Subsequently, countries involved in this study were subjected to a visualization analysis ( Fig. 5 ). The results revealed that the country cooperation network centered around the United States exhibited the largest scale, encompassing 28 links and a total link strength (TLS) of 123, followed by Canada, which had 13 links and a TLS of 66, and England, which had 19 links and a TLS of 58. The size of the node indicates the number of documents published in a specific country, lines represent the frequency of cooperation between countries, and the thickness of the lines depicts the size of the collaboration between the authors or countries. Based on this information, 11 clusters were identified ( Fig. 6 ).

Regarding the Arab country collaboration map, Egypt leads in collaborating in publishing articles with a TLS of 13, followed by Saudi Arabia with a TLS of 12 ( Fig. 6 and Table 2 ).

**Table 2:** Top five countries publishing abdominoplasty research in the Arab region

Country	Number of publications	Number of citations	Total link strength
**Egypt**	30	124	13
**Saudi Arabia**	23	150	12
**Qatar**	11	125	2
**Canada**	8	28	11
**Lebanon**	7	86	5

### Institutions

**Fig. 7:**
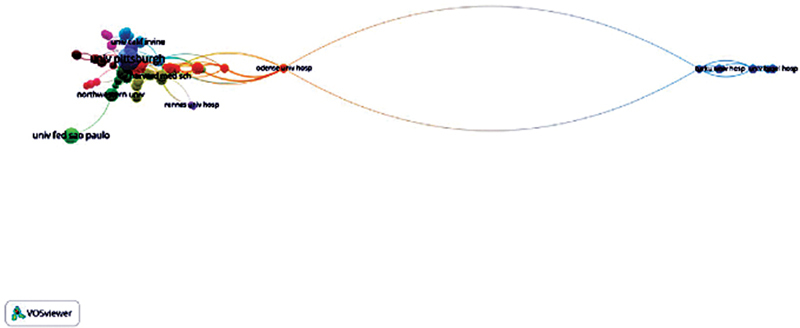
Network visualization map of institution co-authorship worldwide.

Based on the criteria of “minimum number of documents = 5” and visualization analysis, a total of 127 institutions were estimated from 1,884 institutions ( Fig. 7 ). The results revealed that these institutions were primarily divided into 15 clusters ( Table 3 ). The most prolific organization was the University of Pittsburgh (68 publications), followed by Federal University of São Paulo with 31 publications. Also, the University of Pittsburgh had the highest number of citations ( *N* = 1,862), followed by Harvard University ( *N* = 851). Organizations with the highest TLS were McMaster and Pittsburgh universities (TLS = 41 and 28, respectively).

**Table 3:** Top 10 institutions publishing abdominoplasty research

Institution	Publications	Citations
**University Pittsburgh**	68	1,862
**University Fed Sao Paulo**	51	1,036
**Harvard Medical School**	36	1,087
**Northwestern University**	22	444
**Michigan State University**	19	781
**Manhattan Eye, Ear, and Throat Hospital**	19	353
**McMaster University**	18	462
**Stanford University**	17	382
**University of Pennsylvania**	16	356
**University of California**	16	588

**Fig. 8:**
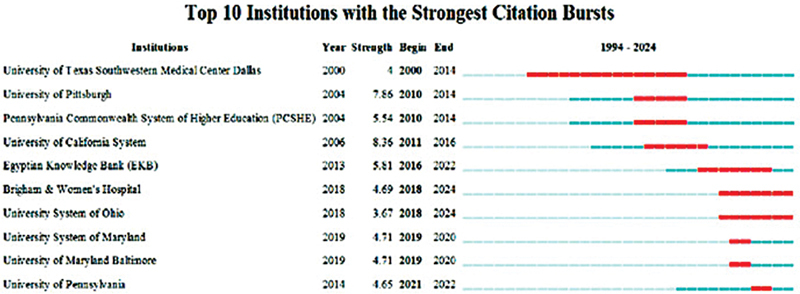
List of top 10 institutions with the strongest citation burst in abdominoplasty.

The Burstness View of CiteSpace displayed the institutions with the strongest citation bursts ( Fig. 8 ). The University of California (with a centrality value of 8.36 from 2011 to 2016) had the strongest citation burst, followed by the University of Pittsburgh (7.86 from 2010 to 2014). University of Texas Southwestern Medical Center, Dallas exhibited the longest burst duration (up to 14 years). Brigham and Women's Hospital and the University of Ohio were the burst institutions from 2018 to January 2024 ( Fig. 8 ).

All publications from Arab countries were distributed among 128 institutions. The leading institutions were the King Saud Medical City ( *N* = 21), Hamad Medical Corporation ( *N* = 9), and Ain Shams University ( *N* = 8). Most of the productive institutions were from Saudi Arabia and Egypt ( Table 4 ).

**Table 4:** Top institutions publishing abdominoplasty research in Arab countries

Institution	Publications	Citations
**King Saud Medical City**	21	110
**Hamad Medical Cooperation**	9	86
**Ain Shams University**	8	26
**King Abdulaziz University**	7	79
**American University of Beirut**	5	25
**Cairo University**	5	26
**Mansoura University**	5	48

### Journals

A total of 297 journals were estimated, of which 40 journals published more than five publications. The leading journals that emerged in this field include Plastic and Reconstructive Surgery ( *N* = 311, 16.4%), Aesthetic Plastic Surgery ( *N* = 241, 12.8%), Annals of Plastic Surgery ( *N* = 166, 8.8%), and Aesthetic Surgery Journal ( *N* = 159, 8.4%). These journals also occupied the top four in terms of total citations. About 66.6% of the articles in the WoSCC online database were published in the top 10 active journals, with a total of 1,257 publications ( Table 5 ). Among the top 10 most productive journals, Plastic and Reconstructive Surgery had the highest Impact Factor (3.2).

**Table 5:** Top 10 journals publishing in abdominoplasty

Journal	Publications	Citations	IF
**Plastic and Reconstructive Surgery**	311	11,514	3.2
**Aesthetic Plastic Surgery**	241	3,523	2
**Annals of Plastic Surgery**	166	3,197	1.4
**Aesthetic Surgery Journal**	159	3,027	3
**Plastic and Reconstructive Surgery-Global Open**	93	596	1.5
**Clinics in Plastic Surgery**	84	1,245	1.8
**Obesity Surgery**	77	1,885	2.9
**Journal of Plastic Reconstructive and Aesthetic Surgery**	72	1,440	2
**European Journal of Plastic Surgery**	32	92	0.6
**Journal of Plastic Surgery and Hand Surgery**	22	294	1

Abbreviation: IF, Impact Factor.

**Fig. 9:**
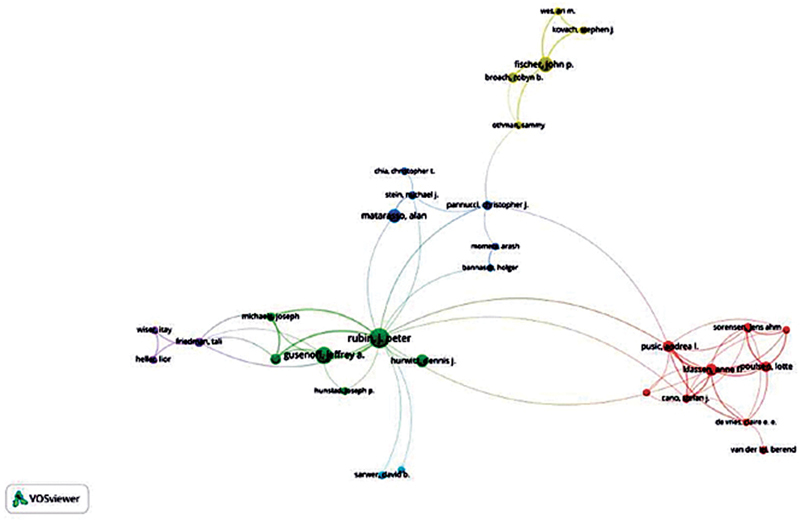
Network visualization map of co-cited journals of abdominoplasty.

According to the visualization information, the most frequently co-cited journal was Plastic and Reconstructive Surgery ( *N* = 11,357; Fig. 9 ), followed by Aesthetic Plastic Surgery ( *N* = 2,906), Aesthetic Surgery Journal ( *N* = 2,819), Annals of Plastic Surgery ( *N* = 2,579), and Obesity Surgery ( *N* = 1,997). Furthermore, most of the highly co-cited journals were from the United States.

In Arab countries, four of the top five journals accepting articles from the Arab area were the same as those identified in the global analysis, namely Aesthetic Plastic Surgery, Plastic and Reconstructive Surgery-Global Open. Plastic and Reconstructive Surgery, and Obesity Surgery ( Table 6 ).

**Table 6:** Top 10 journals publishing in abdominoplasty from Arab countries

Journal	Publications	Citations	IF
**Aesthetic Plastic Surgery**	10	90	2
**Plastic and Reconstructive Surgery-Global Open**	9	9	1.5
**Egyptian Journal of Surgery**	7	4	0.1
**Plastic and Reconstructive Surgery**	5	84	3.2
**Obesity Surgery**	3	9	2.9

Abbreviation: IF, Impact Factor.

### Authors

**Fig. 10:**
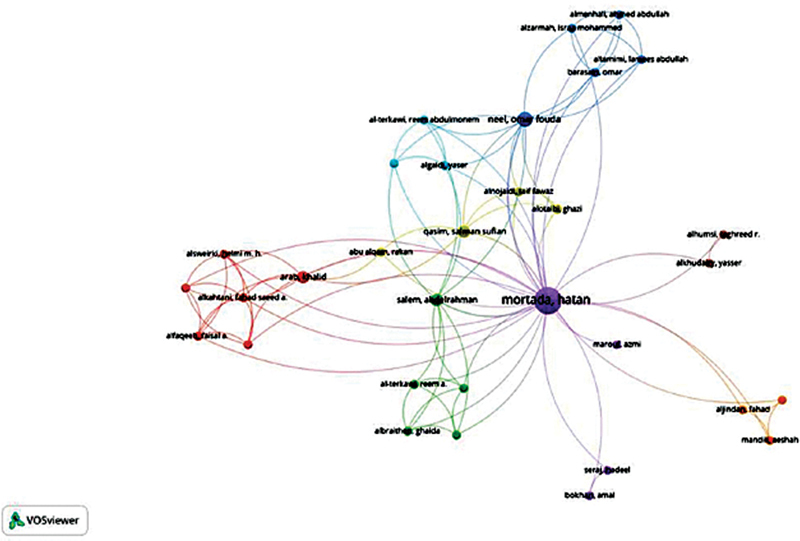
Network map of author co-authorship.

**Fig. 11:**
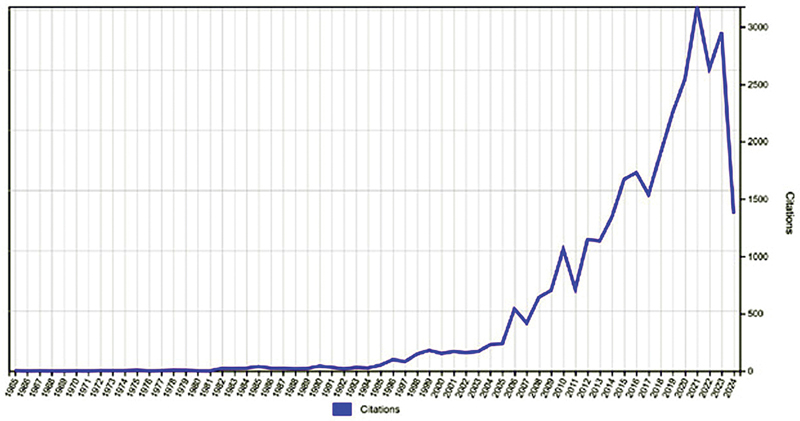
Times cited abdominoplasty publications worldwide.

A total of 6,157 authors had published papers on abdominoplasty. Of them, 111 authors were identified to have published at least five publications. Visualization analysis revealed that 31 authors were interconnected and divided into six main clusters. It is noted that the same color cluster has a strong cooperative relationship between the authors ( Fig. 10 ). In the Arab research output, 358 authors were identified ( Fig. 11 ).

### Analysis of Citation and Co-citation (References)

**Fig. 12:**
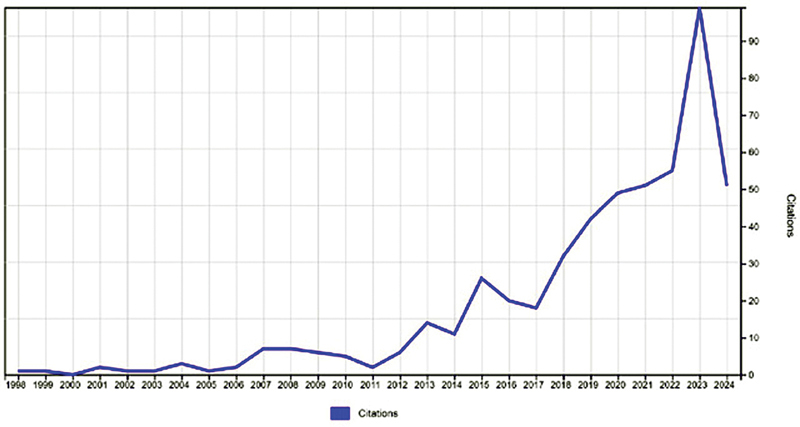
Times cited abdominoplasty publications in Arab countries.

The analyzed publications included a total of 34,781 citations, with an average citation rate of 18.44 citations per article, while approximately 50 publications eclipsed the distinguished landmark of 100 citations. The peak for citation was reached in 2021 ( *N* = 3,422). In both 2022 ( *N* = 2,847) and 2023 ( *N* = 3,157), the number exceeded 2,500 citations ( Fig. 12 ). The majority of these articles found their place in prestigious journals, such as Plastic and Reconstructive Surgery. This highlights the domination of the journal in plastic surgery research, particularly high-quality research. For Arab countries, the retrieved documents received a total of 447 citations, with an average of 6.3 citations per document. The top-most cited documents were identified and are presented in Tables 7 and 8 ).

**Table 7:** The top 10 cited publications in the field of abdominoplasty worldwide

Title	Authors	Source title	Publication year	Total citations
**Free Abdominoplasty Flap and Its Use in Breast Reconstruction - Experimental-Study and Clinical Case-Report**	Holmstrom, H	Scandinavian Journal of Plastic and Reconstructive Surgery and Hand Surgery	1979	353
**Superficial Fascial System (SFS) of the Trunk and Extremities - A New Concept**	Lockwood, Te	Plastic and Reconstructive Surgery	1991	250
**Abdominoplasty Assessed by Survey, with Emphasis on Complications**	Grazer, Fm; Goldwyn, Rm	Plastic and Reconstructive Surgery	1977	226
**Progressive Tension Sutures: A Technique to Reduce Local Complications in Abdominoplasty**	Pollock, H; Pollock, T	Plastic and Reconstructive Surgery	2000	207
**Selective Cryolysis: A Novel Method of Non-Invasive Fat Removal**	Manstein, Dieter; Laubach, Hans; Watanabe, Kanna; Farinelli, William; Zurakowski, David; Anderson, R. Rox	Lasers in Surgery and Medicine	2008	202
**Analysis of Complications from Abdominoplasty - A Review of 206 Cases at a University Hospital**	Neaman, Keith C.; Hansen, Juliana E.	Annals of Plastic Surgery	2007	184
**Body Image and Quality of Life in Post Massive Weight Loss Body Contouring Patients**	Song, Angela Y.; Rubin, J. Peter; Thomas, Veena; Dudas, Jason R.; Marra, Kacey G.; Fernstrom, Madelyn H.	Obesity	2006	173
**Wound Healing Problems in Smokers and Nonsmokers after 132 Abdominoplasties**	Manassa, EH; Hertl, CH; Olbrisch, RR	Plastic and Reconstructive Surgery	2003	164
**High-Lateral-Tension Abdominoplasty with Superficial Fascial System Suspension**	Lockwood, T	Plastic and Reconstructive Surgery	1995	163
**Abdominoplasty: Risk Factors, Complication Rates, and Safety of Combined Procedures**	Winocour, Julian; Gupta, Varun; Ramirez, J. Roberto; Shack, R. Bruce; Grotting, James C.; Higdon, K. Kye	Plastic and Reconstructive Surgery	2015	162

**Table 8:** The top 10 cited publications in the field of abdominoplasty in Arab countries

Title	Authors	Source title	Publication year	Total citations
**Abdominoplasty in Multiparous Women with Severe Musculoaponeurotic Laxity**	Alqattan, MM	British Journal of Plastic Surgery	1997	53
**Does the Addition of Progressive Tension Sutures to Drains Reduce Seroma Incidence after Abdominoplasty? A Systematic Review and Meta-Analysis**	Jabbour, Samer; Awaida, Cyril; Mhawej, Rachad; Habre, Samer Bassilios; Nasr, Marwan	Aesthetic Surgery Journal	2017	44
**Prevalence and Desire for Body Contouring Surgery in Postbariatric Patients in Saudi Arabia**	Aldaqal, Saleh M.; Samargandi, Osama A.; El-Deek, Basem S.; Awan, Basim A.; Ashy, Abdulrahman A.; Kensarah, Ahmed A.	North American Journal of Medical Sciences	2012	35
**Intraabdominal Pressure after Full Abdominoplasty in Obese Multiparous Patients**	Al-Basti, HB; El-Khatib, HA; Taha, A; Sattar, HA; Bener, A	Plastic and Reconstructive Surgery	2004	32
**The Effect of Body Mass Index on Outcome of Abdominoplasty Operations**	Ghnnam, Wagih; Elrahawy, Ashraf; EL Moghazy, Magdy	World Journal of Plastic Surgery	2016	27
**Abdominal Musculoaponeuretic System: Magnetic Resonance Imaging Evaluation before and after Vertical Plication of Rectus Muscle Diastasis in Conjunction with Lipoabdominoplasty**	Elkhatib, Hamdy; Buddhavarapu, Shankar Rao; Henna, Habeba; Kassem, Walid	Plastic and Reconstructive Surgery	2011	26
**Mons Pubis Ptosis: Classification and Strategy for Treatment**	El-Khatib, Hamdy A.	Aesthetic Plastic Surgery	2011	23
**Abdominal Dermolipectomy in an Abdomen with Pre-Existing Scars: A Different Concept**	El-Khatib, HA; Bener, A	Plastic and Reconstructive Surgery	2004	22
**Complications of Body Contouring Surgery in Postbariatric Patients: A Systematic Review and Meta-Analysis**	Marouf, Azmi; Mortada, Hatan	Aesthetic Plastic Surgery	2021	18
**Ultrasound Cavitation Versus Cryolipolysis for Non-Invasive Body Contouring**	Eldesoky, Mohamed Taher Mahmoud; Abutaleb, Enas Elsayed Mohamed; Mousa, Gihan Samir Mohamed	Australasian Journal of Dermatology	2016	18

**Fig. 13:**
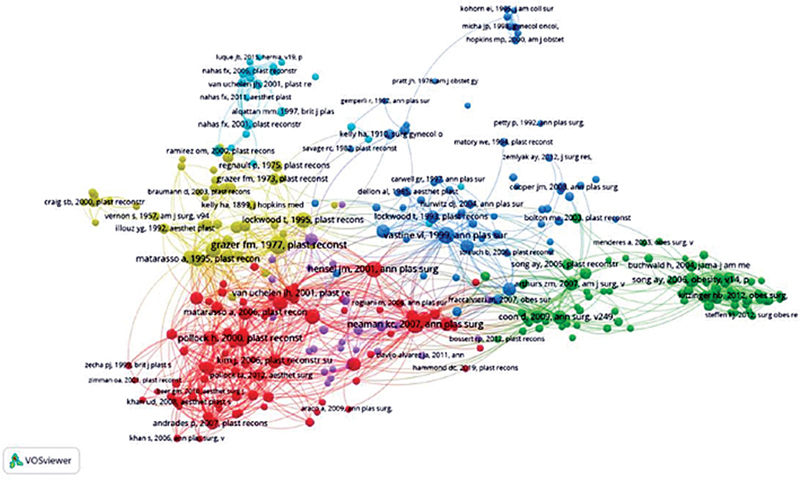
Network visualization map of co-cited references.

The citation network of the most influential scholarly output was analyzed using VOSviewer. The co-cited references that exceeded a threshold of a minimum of 20 citations ( *N* = 313) were identified and represented in 6 clusters to construct a co-citation network visualization ( Fig. 13 ).

### Keywords

In total, 3,845 keywords were identified. Globally, the most prevalent keywords observed were abdominoplasty ( *N* = 792), complications ( *N* = 369), liposuction ( *N* = 233), body contouring ( *N* = 226), bariatric surgery ( *N* = 221), weight loss ( *N* = 217), obesity ( *N* = 185), outcomes ( *N* = 154), panniculectomy ( *N* = 141), and quality of life ( *N* = 127). Similarly, among 362 total keywords from the Arab countries, the top keywords with the most occurrences were analogous to the global trends ( Table 9 ).

**Table 9:** Abdominoplasty research keywords' occurrence globally and in Arab countries

Global research		Arab countries research	
Keyword	Occurrences	Keyword	Occurrences
**Abdominoplasty**	792	**Abdominoplasty**	41
**Complications**	369	**body contouring**	19
**Liposuction**	233	**Weight loss**	19
**Body contouring**	226	**Complications**	16
**Bariatric surgery**	221	**bariatric surgery**	14
**Weight loss**	217	**Liposuction**	12
**Obesity**	185	**Obesity**	9
**Outcomes**	154	**Outcomes**	9
**Panniculectomy**	141	**Efficacy**	7
**Quality of life**	127	**Gastric bypass**	7

**Fig. 14:**
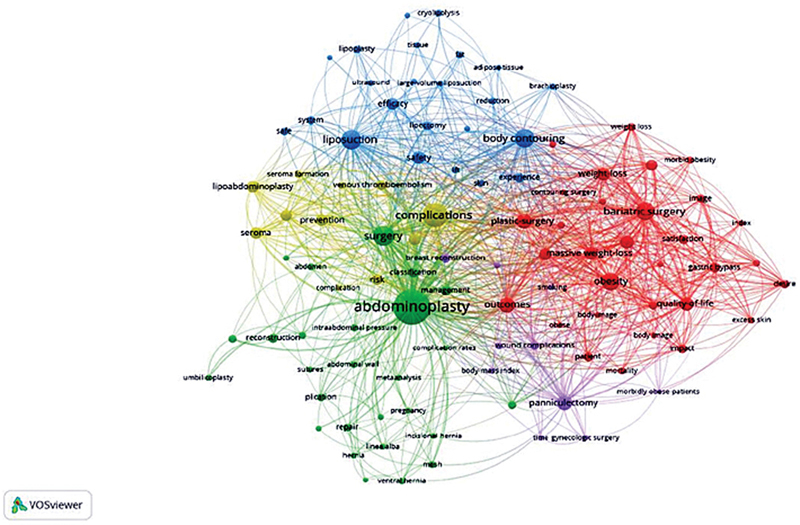
Network visualization map of keyword co-occurrence.

Using VOSviewer and setting a limit of 5 occurrences, 102 exceptional keywords were extracted from the large corpus of 3,845 keywords. The keywords were grouped into five clusters denoting the main research themes. Specifically, the red group mainly describes quality of life, satisfaction, impact, and body image. The green group mainly focuses on abdominoplasty, plication, umbilicoplasty, and reconstruction. The blue group investigates liposuction, lipoplasty, and suction lipectomy. The yellow group examines complications, risk factors, and prevention, while keywords in the purple group mainly explore panniculectomy and gynecologic surgery ( Fig. 14 ).

### Source of Funding

**Fig. 15:**
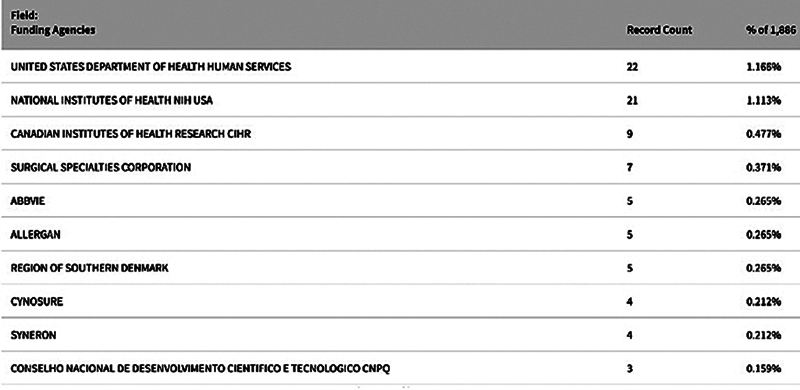
Source of funding.

Most of the identified publications did not report a source of funding. However, for those that did, most were funded by the United States Department of Health and Human Services, National Institute of Health, United States, and the Canadian Institute of Health Research. Similarly, most of the published articles in the Eastern Mediterranean region were not funded ( Fig. 15 ).

### Publications by Subject Category

**Fig. 16:**
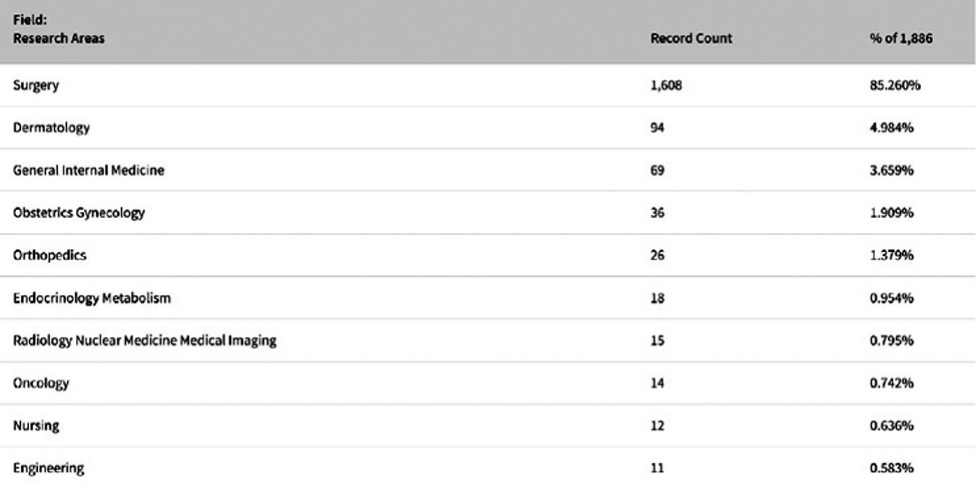
Publication by category.

Most of the retrieved publications were categorized under surgery. Others were categorized under dermatology and general internal medicine, respectively. In research from the Eastern Mediterranean region, surgery was the most common research area, followed by general internal medicine ( Fig. 16 ).

## Discussion

As mentioned before, “… a well-done bibliometric study can build a solid foundation for advancing a field in novel and meaningful ways …”. 16

The outputs of bibliometric analyses are not limited to descriptive statistics but also include the survey of countries, institutions, journals, authors, citations, references, and keywords. 17

Data were gathered from scholarly publications through the Web of Science (WoSCC) database. The current research necessitates the procurement of scholarly literature from the WoSCC database. Subsequently, data were analyzed via visual methodologies using VOSviewer, which developed intricate visualization methodologies, enabled by deploying variable data. 14

The scope of this study covers the period from 1960 to January 2024. In 1960, the modern traditional abdominoplasty was introduced. Yet, modifications of the technique, assessment of risk factors and measurement of outcome, patient satisfaction, and quality of life are still evolving. 18 19

A bibliometric study from Shanghai (from January 1, 2011 to December 31, 2021) analyzed abdominoplasty-related publications and 1,119 articles were included. The search keyword was abdominoplasty. Non-English studies, letters, case reports, book chapters, and withdrawals were excluded. 20

In this work, abdominoplasty, tummy tuck, panniculectomy, and body contour were used as keywords and 1,886 publications finally met our criteria. Both studies used WoSCC as the search engine.

It is clear that researchers have been publishing on the topic since 1960, but the actual pace of publication began to increase after 1993. There was a marked increase in the number of publications from 2014 to 2024. Meanwhile, Arab countries showed improvement in the number of publications from 2010 after a long period of paucity and fluctuation in the number of publications.

United States surpasses other countries with regard to publications, citations, and TLSs. This is not surprising for United States, which has dominance in other medical fields such as stem cells, orthopaedics, and obstetrics. Also, it engages in numerous international collaborations with other countries, intensifying its impact in the scientific field. 21 22 23

In Arab nations, the enduring economic and political instability in some Arab countries could be the reason for their lack of contributions to abdominoplasty research output. Egypt and Saudi Arabia dominate publications. Egypt is the most populous country in the region, with a population estimated to be more than 110 million people in 2024 and Saudi Arabia's gross domestic product exceeds $1 trillion (2023 World Bank). 24 It is interesting to note that Qatar had 125 citations with only 11 published documents in abdominoplasty.

Again, most of the top 10 institutions with citation bursts belong to the United States. This reflects the solid role of its institutions and their influential role in abdominoplasty. Likewise, Saudi Arabia has a leading role in the Arab world, followed by Egypt, Qatar, and Lebanon.

Identifying the prolific journals with higher citations can help scholars in improving their scientific and clinical knowledge and understanding the recent updates and future research trajectories. On analyzing journal hierarchies, the top three journals were the Plastic and Reconstructive Surgery journal, Aesthetic Plastic Surgery, and Annals of Plastic Surgery. Notably, the Plastic and Reconstructive Surgery had 11,514 total citations, exceeding those of Aesthetic Plastic Surgery ( *N* = 3523) and Annals of Plastic Surgery ( *N* = 3,197).

In Arab countries, Aesthetic Plastic Surgery and Plastic and Reconstructive Surgery were the top leading journals in terms of publications and aggregation of citations.

The leading authors of abdominoplasty were from the United States. Rubin, J. Peter, in one of his publications, addressed that Fleur-de-lis abdominoplasty was used to contour the abdomen after massive weight loss. It could be associated with the correction of mons and lower body lift. Special precautions should be taken for patients with high body mass index (BMI) and comorbidities. 25

Focusing on the Arab realm, Hatan Mortada from King Saud University published a systematic review of complications after postbariatric body contouring surgeries. He concluded that when BMI was >30 kg/m ^2^ , the risk of complications increased (37%), and the greater the amount of the resected tissue, the higher the postoperative complications. 26

Citation count has been used in academia as an indicator of the impact of an article as well as an indicator of the quality of the research. 27 As regards the most cited papers, beginning from 1960s, researchers from Chicago University and Calcutta University of India (8 citations) conducted a study to improve the results of panniculectomy by applying a twill tape suture as a guide for skin incision. A mechanical hydraulic lift was used to keep the flap elevated during the operation and the wound was drained by a Hemovac drain. 28

During the 1970s, an article from Sweden (352 citations) reported an experimental and clinical case report study of two cases. Hans Holmström used a free abdominal skin flap after abdominoplasty for breast reconstruction. The donor site was closed in the bikini area. The first case failed due to venous thrombosis. The other case succeeded after anastomosis of three veins. 29

In the 1980s, a review article from Atlanta, Georgia, United States was the highly cited paper (123 citations). Of 563 patients who underwent abdominoplasty, 117 patients underwent abdominoplasty alone, while the rest had the procedure combined with other intra-abdominal or intrapelvic operations without any other aesthetic surgical procedure. The major complications were death, pulmonary embolism, and infection. The major risk factor was obesity and not the complexity of the procedure. 30

Lockwood from the United States, with 248 citations, was the author of the most cited article in the 1990s. He studied the superficial fascial system (SFS) in 12 cadavers and repaired the SFS in 20 patients. The topographic features of the human body relied mainly on SFS. The SFS varied with age, sex, adiposity, and body regions. He emphasized that repair of the SFS of the abdominal area has the same importance as the superficial musculoaponeurotic system repair in rhytidectomy. 31

Due to multiple complications after abdominoplasty, such as hematoma, seroma formation, flap necrosis, and hypertrophic scar, researchers from the Texas group used progressive tension sutures that permitted extensive liposuction combined with abdominoplasty with minimal drawbacks. This study was the most cited article in the 2000s (202 citations). 32

From 2011 to 2020, two articles received the same number of citations (155). Both investigated postoperative complications following abdominoplasty. The first one was conducted at four different places in the United States. A clinical protocol was adopted for patients with a Caprini score of 3 or higher. Patients received postoperative enoxaparin for prophylaxis from venous thromboembolism. They concluded that postoperative enoxaparin prophylaxis was protective against venous thromboembolism. 33 In the second paper, 25,478 abdominoplasties were performed. The overall rate of complications was 4%. The most common complications were hematoma, infection, and venous thromboembolism. Significant risk factors were male ≥55 years of age and BMI ≥30. When abdominoplasties were combined with other procedures such as liposuction, the risk of complications increased. They reached the consensus that abdominoplasty has a higher rate of complications in comparison to other aesthetic procedures. 34

From 2021 to January 2024, a review study from Ohio, United States, with 31 citations showed that the area for fat removal was located between the inframammary fold and the gluteal fold. Liposuction was considered an essential adjunct procedure to boost abdominoplasty results. 35

From the Arabic research community, an article published by Al Qattan, with 52 citations, described 20 multiparous women who underwent abdominoplasty. The type of abdomen presented in this article was the most severe type (resembling that of a full-term pregnancy). 36

It is important to stress that the postoperative results of abdominoplasty of most Arabic women resemble the preoperative images for women in Western countries seeking abdominoplasty. Lack of outdoor activities and limited ability to engage in daily exercise play a role. There is a great need to propose a special classification for abdominal topography in the Arab world.

To anticipate the direction of future research and study in the field of abdominoplasty, it is crucial to examine the hidden relationships among the most prominent publications. One way to do this is by using co-citation networks. This technique helps researchers simplify the complex nature of cross-citations into a more comprehensible visual form. 37 When two references are cited by a literature at the same time, they constitute a co-citation relationship, and the higher the number of co-citations, the more similar the research directions of the two cited works are. The most co-cited reference was “Abdominoplasty assessed by survey, with emphasis on complications.” In this paper, a survey was conducted by 958 researchers to identify abdominoplasty complications. 38

Generally, the title of a paper gives an idea about the study. However, keywords highlight areas of hotspots, research development, trends, and frontiers in research. 39

The results of keyword and reference clustering analysis highlight the primary areas of interest for researchers. By analyzing keywords, we succinctly summarize research hotspots into five distinct periods. From 1960 to 1999, the interest was on surgical technique (e.g., abdominal wall reconstruction, abdominoplasty, experience, lipoabdominoplasty, …). In the following period (2000–2010), the era of bariatric surgery and body contouring surgery appeared. Keywords such as gastric bypass, morbid obesity, and body contouring were common. The third period (2011–2015) focused on postoperative morbidity (e.g., seroma, venous thromboembolism, panniculectomy). The next period (2016–2020) was dedicated to identifying risk factors, minimizing complications, and improving patient satisfaction (risk factors, satisfaction, and outcome). From 2021 to January 2024, there was a return to techniques but with updated modifications (repair, ventral hernia). Actually, abdominoplasty is expected to continue evolving in the forthcoming years.

Funding seems to play a direct or indirect role not only in increasing scientific publications but also in the formation of research teams and international collaboration. 40

Several articles have highlighted the pivotal role of funding in enhancing research productivity. So, the leading role of the United States, Canada, Europe, and other wealthier nations in seminal activities could be explained. 41 Other countries experiencing financial crisis, conflicts, or regional wars consider research as a sort of luxury. Furthermore, these countries fall into the low- to middle-income countries and low-income countries where research funding is limited, with a limited number of researchers. Egypt's expenses on scientific research do not exceed 1% of its national budget. 42

It is evident that the dominance of plastic surgery in abdominoplasty is still unchallenged. Plastic surgeries and plastic surgeons still attract a large number of viewers on the internet. 43 Perhaps the competition is more evident in the subspecialty of facial surgery, where otolaryngology, dermatology, and ophthalmology play significant roles. 44

This study provides a comprehensive analysis of the publication output of abdominoplasty based on a large number of research articles and citations, which excludes the potential bias that may occur in subjective evaluations. Also, leading institutions and eminent authors were identified in the field to help investigators and surgeons in identifying potential training places and experts. Additionally, the results of reference and keyword analyses highlight the key research areas and the future directions of development in this field. Furthermore, shedding light on Arab countries and their contribution in the field adds a new aspect to the study.

However, there are some drawbacks of this research that should be mentioned. First, the data were collected from the WoSCC database. It is a comprehensive and large database site that includes different specialties and disciplines, but not all scientific journals are indexed in WoSCC. Second, due to the limitation of the keyword search terms, some relevant articles may not have been captured. Third, publications were confined to research articles and reviews. Influential studies published in the form of letters, meeting abstracts, and preprints could be overlooked. Also, gray literature was not included, which may have negatively affected the total number of retrieved records. Finally, data obtained from WoSCC reflect the information present in it. Therefore, if an author has more than one profile, their publications might be scattered. The same applies to the names of institutions and countries.

We recommend that future research subcategorize abdominoplasty (e.g., by technique, risk factors, complications, and outcomes) for more granular analysis. To address the identified research gap in the Arab world, we also recommend targeted strategies: national health ministries and major hospitals should allocate dedicated research funds for plastic surgery; leading institutions should invest in formal research methodology training to equip surgeons for high-impact publishing; and prominent Arab institutions should foster strategic collaborations with productive international centers to improve local expertise and citation impact. Finally, the formation of an international support network for researchers, especially in developing countries, remains highly recommended.

## Conclusion

This bibliometric analysis provides comprehensive information for a better understanding of the abdominoplasty research landscape, its hotspots, and its frontiers. The data reflect a rapidly expanding field dominated by the United States, but also highlight a significant opportunity for increased contribution from the Arab world. By implementing targeted strategies to enhance research funding, methodology, and international collaboration, researchers in this region can play a more prominent role in this evolving field. This study should galvanize the acumen necessary to understand the multifaceted subjects of abdominoplasty, and it is anticipated that more researchers will perform similar analyses in plastic surgery and other surgical fields.
